# Perceived effectiveness of messages to address cervical cancer screening barriers: An online experiment

**DOI:** 10.1371/journal.pone.0336693

**Published:** 2025-11-14

**Authors:** Sarah M. Halvorson-Fried, Isabella C.A. Higgins, Melissa B. Gilkey, Allison J. Lazard, Marissa G. Hall

**Affiliations:** 1 Department of Health Behavior, Gillings School of Global Public Health, University of North Carolina at Chapel Hill, Chapel Hill, North Carolina, United States of America; 2 Cancer Prevention and Control, Lineberger Comprehensive Cancer Center, University of North Carolina at Chapel Hill, Chapel Hill, North Carolina, United States of America; 3 Carolina Population Center, University of North Carolina at Chapel Hill, Chapel Hill, North Carolina, United States of America; 4 Hussman School of Journalism and Media, University of North Carolina at Chapel Hill, Chapel Hill, North Carolina, United States of America; University of Birmingham, UNITED KINGDOM OF GREAT BRITAIN AND NORTHERN IRELAND

## Abstract

**Introduction:**

Cervical cancer is almost entirely preventable through vaccination and screening, but screening rates still lag targets. Communication campaigns can encourage screening; however, the types of message content that are most effective are unknown.

**Methods:**

We conducted an online randomized experiment testing messages within four themes aligned with previously identified screening barriers: cancer fatalism, inconvenience, lack of knowledge about risk factors, and unawareness of screening guidelines. A national convenience sample of US participants aged 21–65 years and assigned female at birth (*n* = 1,536) viewed one of three messages from each theme assigned at random and one control message in random order. We measured perceived effectiveness to encourage cervical cancer screening, anticipated social interactions, and self-reported learning. Mixed-effects linear models examined the impact of message theme on each outcome on a scale from 1 (low) to 5 (high).

**Results:**

All four barrier-focused themes encouraged cervical cancer screening more than the control (perceived message effectiveness mean and standard deviation: cancer fatalism = 3.44 (1.21); convenience = 3.43 (1.23); risk factors = 3.25 (1.23); screening guidelines = 3.44 (1.19); control message = 2.45 (1.35), *p* < .001). Barrier-focused messages similarly outperformed the control on anticipated social interactions and self-reported learning (all *p* < .001). Messages were less effective for participants who had never been screened or were out-of-date. However, regardless of screening status, barrier-focused messages outperformed the control.

**Conclusions:**

Messages targeting known barriers to cervical cancer screening were perceived as more effective than a control message. These messages could increase cervical cancer screening rates if used in interventions at scale.

## Introduction

Cervical cancer rates have decreased more than 70% since the 1950s, a success attributed to cervical cancer screening practices [1,2]. Furthermore, with screening and the advent of the human papillomavirus (HPV) vaccine, 93% of cervical cancer cases are now preventable [3]. Despite these advances, nearly 14,000 new cases of cervical cancer and over 4,000 deaths from cervical cancer were expected in the United States (US) in 2024 [4]. Over half of new cervical cancers occur in women who are considered overdue for screening [3]. Despite increased health care access because of the Affordable Care Act [5], the proportion of US women overdue for screening increased more than 8 percentage points from 2005 to 2019 [6]. Furthermore, notable sociodemographic disparities exist in cervical cancer screening, incidence, and mortality rates. Women who are younger, Asian, Native Hawaiian/Pacific Islander, American Indian/Alaska Native, Hispanic, included in “other race” categories, LGBQ + , live in rural areas, have lower education, or do not have health insurance are more likely to be overdue for screening [6–10].

Identified barriers to cervical cancer screening include lack of knowledge or awareness about cervical cancer screening [6,11–13]; financial and logistical barriers such as cost and inconvenience [6,13,14]; and psychosocial barriers such as fear, anxiety, and embarrassment [13–15]. A majority of 2019 National Health Interview Survey participants who were overdue for screening reported that the primary reason they had not been screened was being unaware of screening recommendations or not receiving a recommendation from a provider [6]. This finding suggests that many people may not be adhering to screening guidelines due to lack of awareness. Public health messages can increase knowledge and awareness [16] and have the potential to increase screening rates and decrease the burden of cervical cancer [17]. Such messages could be delivered through text message and social media campaigns, public service announcements, or through medical providers. Previous research suggests that messages can increase cervical cancer screening rates and screening intentions regardless of loss or gain framing (i.e., placing message emphasis on potential losses from not being screened vs. potential gains from being screened) [18,19]. In addition, a recent online experiment found that videos focused on knowledge barriers about Pap tests were perceived as more effective than videos focused on psychological barriers [20]. However, no studies have examined potential differences in effectiveness among a wider variety of message themes, including additional types of barriers that messages can address.

In this study, we aimed to fill this gap by testing messages designed to address four identified barriers to cervical cancer screening: cancer fatalism (the belief that developing cancer is out of one’s control [21]), inconvenience, lack of knowledge of risk factors, and unawareness of screening guidelines. We aimed to examine the *perceived message effectiveness,* or how much the message encourages screening, of each message theme compared to a control message. Perceived message effectiveness is both predictive of behavior change [22,23] and sensitive to small differences among messages [24], which is helpful because different messages can be compared. We also examined anticipated social interactions (the likelihood of discussing message content with others) and self-reported learning (the extent to which the message taught the participant something they did not already know), two potential mechanisms by which messages may influence individuals to receive screening. In addition, we examined whether cervical cancer screening status moderated perceived effectiveness. Finally, we examined sociodemographic predictors of perceived effectiveness to examine if certain groups were more responsive to messages overall.

## Methods

### Message development

To develop messages for the experiment, we first reviewed the literature on key barriers to cervical cancer screening uptake and adherence. We focused on barriers that can be addressed at the individual level rather than structural barriers (e.g., no insurance coverage). The four key barriers identified included cancer fatalism, inconvenience, lack of knowledge about risk factors for cervical cancer, and lack of awareness of screening guidelines [25–30]. We developed three messages for each barrier (Table 1) and one general statement for the control (“Schedule your screening today”).

**Table 1:** Unadjusted perceived message effectiveness by single message within each barrier-focused theme (n = 1,536).

	Mean	SD
**Control Message**		
Schedule your screening today.	2.45	1.35
**Cancer Fatalism**		
Your health is in your hands. With routine cervical cancer screening, you can stop cancer before it starts. Schedule your screening today.	3.63	1.13
Good news! Cervical cancer screening can prevent cervical cancer. Schedule your screening today.	3.26	1.26
There is a plan for your life, and it doesn’t include cervical cancer. With routine cervical cancer screening, you can prevent cervical cancer. Schedule your screening today.	3.43	1.22
**Convenience**		
When it’s time to get checked out, don’t put it off. Cervical cancer screening is quick and easy. Schedule your screening today.	3.27	1.26
Imagine losing your life to cancer because you didn’t make time for a quick cervical cancer screening. Schedule your screening today.	3.59	1.23
No time for cancer treatment? Then make time for cancer prevention. Schedule your screening today.	3.43	1.17
**Risk Factors**		
Cervical cancer screening is important even if you are not sexually active. Schedule your screening today.	3.35	1.20
It doesn’t matter if you’re monogamous or into hookups. You’re still at risk for cervical cancer. Schedule your screening today.	3.12	1.27
Even if you received the HPV vaccine, it’s still important to get routine cervical cancer screening. Schedule your screening today.	3.29	1.22
**Screening Guidelines**		
Did you know? It is recommended to get routine cervical cancer screening from ages 21–65 to prevent cancer. Schedule your screening today.	3.48	1.16
Did you know? Doctors recommend everyone with a cervix receive routine cervical cancer screening from ages 21–65 to prevent cancer. Schedule your screening today.	3.46	1.24
Did you know? Everyone with a cervix should receive routine cervical cancer screening from ages 21–65. Schedule your screening today.	3.40	1.17

*Notes.* SD = standard deviation.

### Participants and procedures

We conducted an online experiment as a supplement to a study that primarily tested the impacts of taxes and warning labels on red meat purchases, which have been linked to cancer and cardiovascular disease [31] (i.e., the “parent study”) [32]. Participants were a convenience sample recruited from Cloud Research Prime Panels, a survey research firm commonly used for social science research. Participants were eligible for the parent study if they were aged 18 years or older, resided in the United States, and reported eating red meat at least once per week during the past 30 days and doing at least 50% of the grocery shopping for their household [32]. Participants were additionally eligible to participate in the current study about cervical cancer screening messages if they were assigned female at birth and aged 21–65, since screening is recommended every 3 years for people with cervixes aged 21–29 and every 5 years for those aged 30–65 [33]. For the current study, 1,587 individuals completed the experimental task; 51 were excluded from analysis because after inclusion they reported not having a cervix due to surgery, resulting in an analytic sample of 1,536 participants.

After completing all sections of the parent study survey, except for the sociodemographic section, participants were screened for eligibility for the current study. Those who were eligible viewed and rated five messages: one of three messages from each of the four barrier-focused message themes (randomly selected from each theme) and one control message. All messages were displayed in random order and were presented as black, centered text on a white screen. Participants then completed the sociodemographic section of the survey. Recruitment and data collection occurred from March 11 to October 21, 2021.

### Measures

The primary outcome for this study was perceived message effectiveness, which we measured with one survey item: “How much does this message encourage you to get screened for cervical cancer?” with five response options: not at all (coded as 1), a little bit (2), somewhat (3), quite a bit (4), and very much (5). We assessed two secondary outcomes: anticipated social interactions and self-reported learning, which are mechanisms by which public health campaigns can affect behavior [34–36]. The survey measured anticipated social interactions with the question “How likely are you to talk about this message with others in the next week?” Response options were not at all likely (coded as 1), a little likely (2), somewhat likely (3), very likely (4), and extremely likely (5). The survey assessed self-reported learning with the question “How much did you learn something new from this message that you did not already know?” Response options were not at all (coded as 1), a little bit (2), somewhat (3), quite a bit (4), and very much (5) [37].

The survey measured sociodemographic characteristics, including cervical cancer screening status, baseline screening intentions, sex, gender, age, race, income, sexual orientation, education, self-reported health, usual place of care, usual provider, insurance status, and cancer fatalism, using survey items developed in previous research [11,38–43]. In addition, we asked participants about reasons for not being up to date with screening (S1 Table) and their desired channel for cervical cancer screening messages (S2 Table).

### Ethics

This study was approved by the Institutional Review Board at the University of North Carolina at Chapel Hill (protocol 19–3349). Participants provided written informed consent. Participants received cash, gift cards, or reward points as incentives for completing the study.

### Statistical analysis

Analyses used Stata version 18 [44] (College Station, TX) and a two-sided alpha value of 0.05. We used mixed-effects linear models with the control as the reference group to examine the impact of message theme (versus control) on the primary and secondary outcomes to account for repeated measures. We estimated separate models for each outcome. We then used postestimation commands to compare the effects of the message themes to each other for all outcomes.

Next, we used a mixed-effects multivariable linear model to examine whether barrier-focused messages remained effective when controlling for person-level characteristics. Person-level characteristics included in the model were age, race/ethnicity, health insurance status, income, educational attainment, sexual orientation, self-reported health, having a usual medical provider, baseline cancer fatalism, screening status within the past five years, and baseline screening intentions. Predictors were chosen based on associations with screening rates [6–8]. For education, race, income, sexual orientation, self-reported health, having a usual provider, and health insurance status, we collapsed categories for interpretability or because of small cell sizes. Though we planned to include gender in the model, we dropped this variable because over 99% of the sample identified as women. We used listwise deletion to handle missing data.

Finally, we examined whether the perceived effectiveness of each barrier-focused theme was moderated by having had a cervical cancer screening within the past five years and having ever had a screening. For these analyses, we ran two separate mixed-effects linear models (one for each moderator), regressing perceived message effectiveness on message theme, the moderator, and the interaction of message theme with the moderator. We used a Wald chunk test to test the statistical significance of the interaction term. Analyses were conducted in 2024.

Study predictions, measures, and the analytic plan were pre-registered prior to data collection at AsPredicted.org (protocol 58390).

## Results

The average age of participants was 43.4 years old (Table 2). Most participants identified as White (70%), while 11% identified as Black, 10% as Latine, 4% as Asian, and 5% as another race or multiracial. Around one-third (36%) had attained a high school education or lower, 24% had some college, and 40% had a bachelor’s degree or higher. Approximately one-fifth (22%) of participants reported that they had never been screened for cervical cancer and nearly one-third (32%) reported that they had not received a screening in the past five years, meaning they were not up to date with screening. Almost half (48%) reported that they would definitely get screened if recommended. Most participants had a usual health care provider (81%) and health insurance (90%). Missing data ranged from 0% (0 participants) to 1.95% (30 participants).

**Table 2:** Participant characteristics (n = 1,536).

	n	%
Age, in years (mean [SD])	43.4	13.2
Education		
Less than or completed high school/GED	547	36
Some College	370	24
Bachelors or Grad Degree	617	40
Gender		
Woman	1,526	99
Man, non-binary, or two spirit	10	1
Has had a cervical cancer screening		
Yes	1,199	78
No	318	21
Unsure	17	1
Cervical cancer screening within 5 years		
Yes	1,018	68
No	488	32
Likelihood of getting screened if recommended		
Definitely won’t	84	5
Probably won’t	169	11
Probably will	538	35
Definitely will	745	49
Race/ethnicity		
White, non-Latine	1,079	70
Black, non-Latine	169	11
Latine	154	10
Asian	60	4
Another race or multiracial	70	5
Income		
Less than $25,000	290	19
$25,000-49,999	439	29
$50,000-99,999	548	36
$100,000 or more	255	17
Sexual orientation		
Straight	1,350	89
Lesbian, gay, bisexual, or another orientation	168	11
Self-reported health		
Poor or fair	336	22
Good	687	45
Very good or excellent	509	33
Has a usual care provider		
Yes	1,236	81
No	296	19
Insured		
Yes	1,371	90
No	160	10
Cancer fatalism (mean [SD])	2.35	1.11

*Notes. *SD = standard deviation. Missing data ranged from 0% to 1.95%. Cancer fatalism was measured with a single item: “There’s not much you can do to lower your chances of getting cancer” with Likert response options ranging from 1 (“Strongly Disagree”) to 5 (“Strongly Agree”); item from Health Information and National Trends Survey (HINTS) [43].

### Impact of barrier-focused message themes on primary and secondary outcomes

**Fig 1:**
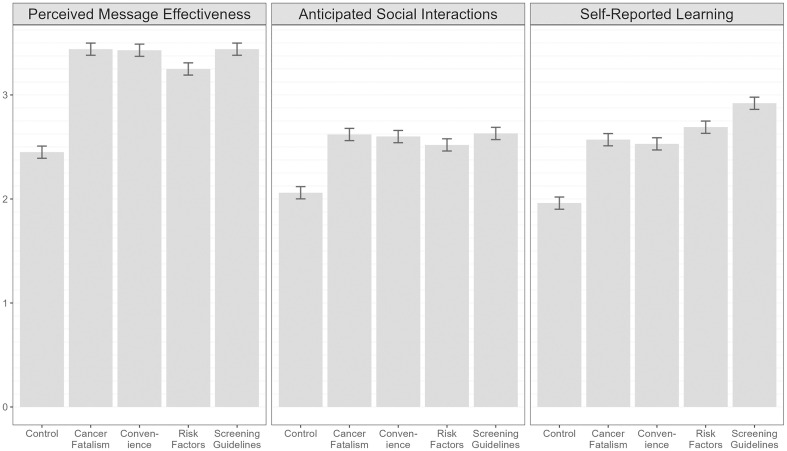
Perceived message effectiveness, anticipated social interactions, and self-reported learning by message theme (n = 1,536). Error bars indicate 95% confidence intervals. Estimates are adjusted for arm in parent study.

All barrier-focused message themes were more encouraging of cervical cancer screening than the control message (mean perceived message effectiveness range 3.25–3.44 versus 2.45, all *p*s < .001) (**Fig 1**). In addition, all barrier-focused message themes elicited higher anticipated social interactions than the control (mean range 2.52–2.63 versus 2.06, all *p*s < . 001) and higher self-reported learning (mean range 2.53–2.92 versus 1.96, all *p*s < .001). Full unadjusted results can be found in S3 Table and perceived effectiveness of each individual message can be found in Table 1.

In pairwise comparisons, messages in the cancer fatalism, convenience, and screening guidelines themes elicited higher perceived message effectiveness than those in the risk factors theme (mean range 3.43–3.44 versus 3.25, all *p*s < .001) (Table 3). The cancer fatalism, convenience, and screening guidelines themes also elicited higher anticipated social interactions than the risk factors theme (2.60–2.63 vs. 2.52, all *p*s < .001). However, messages in the screening guidelines and risk factors themes elicited higher self-reported learning than cancer fatalism and convenience (2.69–2.92 versus 2.53–2.57, all *p*s < .001). Messages focused on screening guidelines elicited higher self-reported learning than those focused on risk factors (2.92 vs. 2.69, *p* < .001). In the multivariable regression model, all barrier-focused message themes led to significantly higher perceived effectiveness than the control message when controlling for sociodemographic characteristics (*β* range 0.81–1.01, all *p*s < .001) (S4 Table).

**Table 3:** Results from all pairwise comparisons (n = 1,536).

Comparison	Beta (95% CI)	P-value	Predicted mean (95% CI)
**Perceived message effectiveness**			
Control (ref)	---	---	2.45 (2.39, 2.52)
Cancer fatalism	0.99 (0.93, 1.04)	**<.001**	3.44 (3.38, 3.50)
Screening	0.99 (0.94, 1.04)	**<.001**	3.44 (3.38, 3.51)
Risk factors	0.80 (0.75, 0.85)	**<.001**	3.25 (3.19, 3.32)
Convenience	0.98 (0.92, 1.03)	**<.001**	3.43 (3.37, 3.49)
Cancer fatalism (ref)	---	---	
Screening	0.00 (−0.05, 0.06)	0.923	
Risk factors	−0.19 (−0.24, −0.14)	**<.001**	
Convenience	−0.01 (−0.06, 0.04)	0.701	
Screening (ref)	---	---	
Risk factors	−0.19 (−0.24, −0.14)	**<.001**	
Convenience	−0.01 (−0.07, 0.04)	0.631	
Risk factors (ref)	---	---	
Convenience	0.18 (0.12, 0.23)	**<.001**	
**Anticipated social interactions**			
Control (ref)	---	---	2.06 (2.00, 2.13)
Cancer fatalism	0.55 (0.51, 0.60)	**<.001**	2.62 (2.55, 2.69)
Screening	0.57 (0.52, 0.61)	**<.001**	2.63 (2.56, 2.70)
Risk factors	0.46 (0.41, 0.50)	**<.001**	2.52 (2.45, 2.59)
Convenience	0.54 (0.49, 0.58)	**<.001**	2.60 (2.53, 2.67)
Cancer fatalism (ref)	---	---	
Screening	0.01 (−0.03, 0.06)	0.553	
Risk factors	−0.10 (−0.14, −0.05)	**<.001**	
Convenience	−0.02 (−0.06, 0.03)	0.501	
Screening (ref)	---	---	
Risk factors	−0.11 (−0.15, −0.06)	**<.001**	
Convenience	−0.03 (−0.07, 0.02)	0.205	
Risk factors (ref)	---	---	
Convenience	0.08 (0.04, 0.13)	**<.001**	
**Self-reported learning**			
Control (ref)	---		1.96 (1.89, 2.03)
Cancer fatalism	0.61 (0.56, 0.67)	**<.001**	2.57 (2.51, 2.64)
Screening	0.96 (0.91, 1.01)	**<.001**	2.92 (2.85, 2.98)
Risk factors	0.73 (0.67, 0.78)	**<.001**	2.69 (2.62, 2.75)
Convenience	0.57 (0.52, 0.62)	**<.001**	2.53 (2.46, 2.60)
Cancer fatalism (ref)	---		
Screening	0.34 (0.29, 0.40)	**<.001**	
Risk factors	0.11 (0.06, 0.17)	**<.001**	
Convenience	−0.04 (−0.09, 0.01)	0.125	
Screening (ref)	---		
Risk factors	−0.23 (−0.28, −0.18)	**<.001**	
Convenience	−0.39 (−0.44, −0.33)	**<.001**	
Risk factors (ref)	---		
Convenience	−0.15 (−0.21, −0.10)	**<.001**	

*Notes.* CI = confidence interval. Predicted means by theme remain consistent across comparisons and thus are only reported in the first section for each outcome. Boldface indicates statistical significance (*p* < .05).

### Moderation by screening status

**Fig 2:**
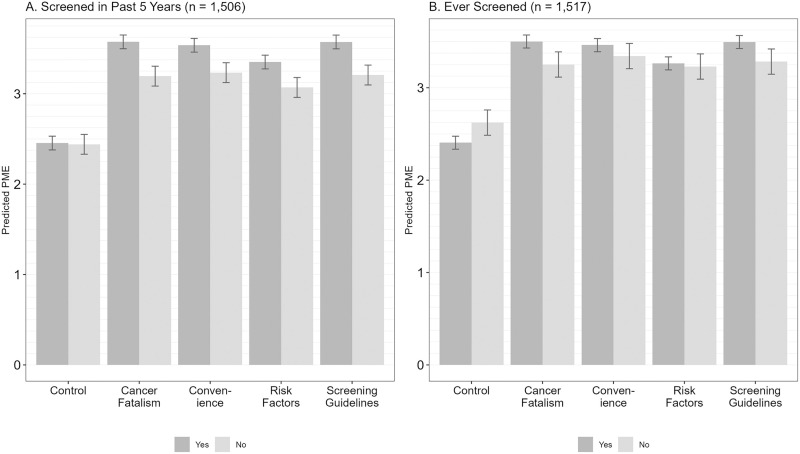
Perceived message effectiveness by screening status. Error bars indicate 95% confidence intervals. Wald chunk test. *p* < .001. PME = perceived message effectiveness.

All barrier-focused message themes outperformed the control message regardless of participants’ screening status. However, barrier-focused messages were less effective for participants who were out-of-date with screening or had never been screened compared to those who were up to date or had been screened before (interaction term *p*s < .001, **Fig 2**). All barrier-focused themes were perceived as less effective by those who were *out-of-date with screening*; perceived effectiveness of the control message was similar for both groups (**Fig 2A**). Participants who had *never been screened* rated the messages focused on cancer fatalism and screening guidelines as less effective and the control message as more effective than those who had. However, perceived effectiveness did not vary by ever screened status for messages on convenience or risk factors (**Fig 2B**).

### Reasons for out of date screening and desired channels for receiving messages

Participants reported that reasons for not being up to date with screening recommendations were primarily related to lack of awareness (e.g., “I didn’t know that I am supposed to have routine cervical cancer screening”) as well as incorrect understanding of risk factors (e.g., “I am not sexually active” and “I have only 1 sexual partner”) (S1 Table). Regarding desired channels for receiving messages, 72% of participants indicated that they would like to receive messages from their doctor, 49% indicated social media, 44% television, 23% magazines, newspapers, billboards, or posters, and 21% indicated their family (S2 Table).

## Discussion

We examined the effectiveness of messages addressing barriers to cervical cancer screening among a sample of US adults recommended for routine cervical cancer screening. All barrier-focused message themes—cancer fatalism, convenience, risk factors, and screening guidelines—encouraged cervical cancer screening more than a generic control message (“Schedule your screening today”). All barrier-focused themes were perceived as more effective compared with the control in both unadjusted models and models that controlled for sociodemographic factors. In addition, all barrier-focused themes elicited higher anticipated social interactions and more self-reported learning than the control. Messages focused on increasing knowledge of risk factors were associated with lower perceived effectiveness and anticipated social interactions than messages in the other themes, but higher self-reported learning. Messages focused on increasing awareness of screening guidelines were associated with the highest self-reported learning.

These findings suggest that messages addressing a variety of screening barriers hold promise for motivating individuals to seek cervical cancer screening. Similar to previous work that has found no difference in effectiveness among messages with gain or loss framing [18,19], we found that messages within all four themes were perceived as effective, with only slight differences in comparisons across themes. This finding suggests that campaigns could use a variety of themes to improve cervical cancer screening rates. Given that lack of knowledge or awareness is a primary reason people do not get screened [6,11,12], it is encouraging that messages focused on increasing knowledge of risk factors and awareness of screening guidelines both led to self-reported learning and were perceived by participants as effective. Messages focused on increasing awareness of screening guidelines performed the best out of all message themes across all three outcomes. This finding aligns with a recent study in which TikTok videos addressing knowledge barriers about Pap tests elicited higher perceived message effectiveness than videos addressing pain and discomfort [20]. Taken together, although differences in outcomes between the themes were small, our findings indicate that messages focused on screening guidelines may have co-benefits of informing people and potentially changing their behavior. Cancer fatalism and convenience are also promising themes for inclusion in health communication materials, while risk factor messages that emphasize the importance of screening even for people who are currently low risk may be less effective (e.g., “Even if you received the HPV vaccine, it’s still important to get routine cervical cancer screening”).

Participants in our sample expressed interest in receiving messages from a variety of sources, including medical providers, social media, and traditional media, suggesting the potential scalability of these messages through distinct channels. Campaigns could consider focusing on provider recommendations, which have consistently been associated with increased screening rates [45]; text message campaigns, which have been found to increase rates of different cancer screenings [46]; as well as advertisements on television, social media, and in public places.

Moderation analyses showed that although participants consistently rated barrier-focused messages higher than the control message regardless of screening status, those who were out-of-date for screening perceived all barrier-focused messages as less effective than those who were up to date. Similarly, participants who had never received a screening rated messages focused on cancer fatalism and screening guidelines as less effective than those who had. These findings indicate that messages may be least effective for those who would most benefit from them, mirroring previous work [47,48]. This may be because regardless of an individual’s desire to receive screening, structural barriers (e.g., lack of transportation, lack of health insurance) may prevent them from doing so. Multilevel interventions may be needed to ensure that structural barriers are addressed in tandem with campaigns encouraging individuals to seek screening.

Key strengths of this study are the use of randomization and pre-registration prior to data collection. A key limitation is that we examined *perceived* effectiveness rather than actual effectiveness, limiting our ability to assess the effects of the messages on actual behavior. However, perceived message effectiveness has been found to correlate strongly with intentions and behavior change for other health behaviors [22,23]. Self-reported learning scores could reflect the amount of novel information messages contained, or the amount of knowledge participants already had; we were not able to distinguish between these two possibilities. In addition, we used a convenience sample potentially limiting the generalizability of study findings, though experimental findings in convenience samples tend to mirror the pattern in nationally representative samples [49]. Although we excluded participants from analysis if they reported not having a cervix due to surgery (e.g., hysterectomy), we did not explicitly ask about this and it is possible that people assigned female at birth who no longer have a cervix were included in the sample. Furthermore, our sample did not have enough gender diversity to test for differences by gender identity, which is needed because trans men have lower rates of screening than cis women [50,51]. Future studies should consider including more gender diverse samples or focusing on trans men and non-binary people with cervixes. We also did not measure urbanicity. Given that cervical cancer screening and incidence rates are higher in rural areas [6,8], this should be considered in future studies.

## Conclusions

Multiple types of cervical cancer screening messages that addressed common barriers to screening were perceived to be effective by participants in an online experiment, and influenced anticipated social interactions and self-reported learning, two behavior change mechanisms. Public health messaging campaigns addressing a variety of barriers to cervical cancer screening hold promise for increasing cervical cancer screening uptake and adherence.

## Supporting information

S1 Table Reasons for not being up to date with screening (n = 487). Participants could select more than one response, so percentages do not total 100%.
(DOCX)

S2 Table Preferred channels for receiving messages about cervical cancer screening (n = 1,535). Participants could select more than one response, so percentages do not total 100%.
(DOCX)

S3 Table Unadjusted primary and secondary outcomes for control message and by barrier-focused theme (n = 1,536). SD = standard deviation.
(DOCX)

S4 Table Adjusted impact of barrier-focused message theme on perceived message effectiveness, controlling for person-level characteristics (n = 1,483). Associations are adjusted for arm in parent study (p = 0.168–0.372). Boldface indicates statistical significance (*p* < .05).
(DOCX)

## References

[1] Safaeian M, Solomon D, Castle PE. Cervical cancer prevention - cervical screening: science in evolution. Obstet Gynecol Clin North Am. 2007;34(4):739–60. 18061867 10.1016/j.ogc.2007.09.004 PMC2762353

[2] Pierce Campbell CM, Menezes LJ, Paskett ED, Giuliano AR. Prevention of invasive cervical cancer in the United States: past, present, and future. Cancer Epidemiol Biomarkers Prev. 2012;21(9):1402–8. doi: 10.1158/1055-9965.EPI-11-1158 22556273 PMC3556792

[3] Centers for Disease Control and Prevention. Cervical cancer is preventable. https://www.cdc.gov/vitalsigns/cervical-cancer/index.html. 2020. Accessed 2024 May 29

[4] National Cancer Institute. Cancer of the cervix uteri - cancer stat facts. SEER. https://seer.cancer.gov/statfacts/html/cervix.html. Accessed 2024 May 29

[5] Courtemanche C, Marton J, Ukert B, Yelowitz A, Zapata D. Effects of the Affordable Care Act on health care access and self-assessed health after 3 years. Inq J Med Care Organ Provis Financ. 2018;55:0046958018796361. 10.1177/0046958018796361 PMC6146333 30188235

[6] Suk R, Hong YR, Rajan SS, Xie Z, Zhu Y, Spencer JC. Assessment of US Preventive Services Task Force guideline–concordant cervical cancer screening rates and reasons for underscreening by age, race and ethnicity, sexual orientation, rurality, and insurance, 2005 to 2019. JAMA Netw Open. 2022. 10.1001/jamanetworkopen.2021.43582 PMC8767443 35040970

[7] McDaniel CC, Hallam HH, Cadwallader T, Lee HY, Chou C. Persistent racial disparities in cervical cancer screening with Pap test. Prev Med Rep. 2021;24:101652. 34976700 10.1016/j.pmedr.2021.101652 PMC8684022

[8] Borders TF, Thaxton Wiggins A. Cervical cancer screening rates among rural and urban females, from 2019 to 2022. JAMA Netw Open. 2024;7(6):e2417094. 10.1001/jamanetworkopen.2024.17094 PMC11179126 38874926

[9] Charkhchi P, Schabath MB, Carlos RC. Modifiers of Cancer Screening Prevention Among Sexual and Gender Minorities in the Behavioral Risk Factor Surveillance System. J Am Coll Radiol. 2019;16(4 Pt B):607–20. doi: 10.1016/j.jacr.2019.02.042 30947895

[10] Charlton BM, Corliss HL, Missmer SA, Frazier AL, Rosario M, Kahn JA. Reproductive health screening disparities and sexual orientation in a cohort study of U.S. adolescent and young adult females. J Adolesc Health. 2011;49(5). 10.1016/j.jadohealth.2011.03.013 PMC3200536 22018565

[11] Marlow LAV, Chorley AJ, Haddrell J, Ferrer R, Waller J. Understanding the heterogeneity of cervical cancer screening non-participants: Data from a national sample of British women. Eur J Cancer. 2017;80:30–8. doi: 10.1016/j.ejca.2017.04.017 28535495 PMC5489076

[12] Akinlotan M, Bolin JN, Helduser J, Ojinnaka C, Lichorad A, McClellan D. Cervical Cancer Screening Barriers and Risk Factor Knowledge Among Uninsured Women. J Community Health. 2017;42(4):770–8. doi: 10.1007/s10900-017-0316-9 28155005 PMC5494033

[13] Kirubarajan A, Leung S, Li X, Yau M, Sobel M. Barriers and facilitators for cervical cancer screening among adolescents and young people: a systematic review. BMC Womens Health. 2021 Mar 23;21(1):122. 33757512 10.1186/s12905-021-01264-x PMC7989022

[14] Lyttle NL, Stadelman K. Assessing awareness and knowledge of breast and cervical cancer among Appalachian women. Prev Chronic Dis. 2006;3(4):A125. 16978500 PMC1779289

[15] Young B, Bedford L, Kendrick D, Vedhara K, Robertson JFR, das Nair R. Factors influencing the decision to attend screening for cancer in the UK: a meta-ethnography of qualitative research. J Public Health Oxf Engl. 2018;40(2):315–39. 10.1093/pubmed/fdx026 28486650

[16] Lee HY, Koopmeiners JS, Rhee TG, Raveis VH, Ahluwalia JS. Mobile phone text messaging intervention for cervical cancer screening: changes in knowledge and behavior pre-post intervention. J Med Internet Res. 2014;16(8):e3576. doi: 10.2196/jmir.3576 PMC4180333 25164545

[17] Morrell S, Perez DA, Hardy M, Cotter T, Bishop JF. Outcomes from a mass media campaign to promote cervical screening in NSW, Australia. J Epidemiol Community Health. 2010;64(9):777–83. 19822553 10.1136/jech.2008.084657

[18] Ogden SN, Leskinen EA, Sarma EA, Wainwright JV, Rendle KA. Effects of message framing on cervical cancer screening knowledge and intentions related to primary HPV testing. Cancer Prev Res (Phila). 2021;14(9):839–44. doi: 10.1158/1940-6207.CAPR-20-0622 33846214 PMC8416706

[19] Adonis L, Paramanund J, Basu D, Luiz J. Framing preventive care messaging and cervical cancer screening in a health-insured population in South Africa: implications for population-based communication?. J Health Psychol. 2017;22(11):1365–75. 26888327 10.1177/1359105316628735

[20] Kirkpatrick CE, Lawrie LL. This is what a speculum looks like! Effects of medical instrument demonstration and message framing in pap test videos on social media. Health Commun. 2025;0(0):1–12. 10.1080/10410236.2025.2511733 40455067

[21] Keller KG, Toriola AT, Schneider JK. The relationship between cancer fatalism and education. Cancer Causes Control. 2021;32(2):109–18. doi: 10.1007/s10552-020-01363-4 33151430 PMC8284073

[22] Noar SM, Barker J, Bell T, Yzer M. Does Perceived Message Effectiveness Predict the Actual Effectiveness of Tobacco Education Messages? A Systematic Review and Meta-Analysis. Health Commun. 2020;35(2):148–57. doi: 10.1080/10410236.2018.1547675 30482058 PMC6538475

[23] Ma H, Gottfredson O’Shea N, Kieu T, Rohde JA, Hall MG, Brewer NT. Examining the longitudinal relationship between perceived and actual message effectiveness: a randomized trial. Health Commun. 2024;39(8):1510–9. 37316818 10.1080/10410236.2023.2222459 PMC10719418

[24] Baig SA, Noar SM, Gottfredson NC, Boynton MH, Ribisl KM, Brewer NT. UNC perceived message effectiveness: validation of a brief scale. Ann Behav Med. 2019;53(8):732–42. 30321252 10.1093/abm/kay080 PMC6636889

[25] Fuzzell LN, Perkins RB, Christy SM, Lake PW, Vadaparampil ST. Cervical cancer screening in the United States: challenges and potential solutions for underscreened groups. Prev Med. 2021;144:106400. 33388330 10.1016/j.ypmed.2020.106400

[26] Senkomago V, Greek A, Jackson JE, Thomas CC, Richardson LC, Benard VB. Learning from cervical cancer survivors: an examination of barriers and facilitators to cervical cancer screening among women in the United States. J Prim Care Community Health. 2021;12:21501327211041862. 34486436 10.1177/21501327211041862 PMC8424614

[27] Luft H, Perzan M, Mitchell R, Schmidt A. An integrative literature review of barriers and facilitators to cervical cancer screening among refugee women in the United States. Health Care Women Int. 2021;42(7–9):992–1012. doi: 10.1080/07399332.2020.1803872 32814006

[28] Johnson CE, Mues KE, Mayne SL, Kiblawi AN. Cervical cancer screening among immigrants and ethnic minorities: a systematic review using the Health Belief Model. J Low Genit Tract Dis. 2008;12(3):232–41. doi: 10.1097/LGT.0b013e31815d8d88 18596467

[29] MacKinnon KM, Risica PM, von Ash T, Scharf AL, Lamy EC. Barriers and motivators to women’s cancer screening: A qualitative study of a sample of diverse women. Cancer. 2023;129(S19):3152–61. doi: 10.1002/cncr.34653 37691528

[30] Shin HY, Song SY, Jun JK, Kim KY, Kang P. Barriers and strategies for cervical cancer screening: What do female university students know and want?. PLoS One. 2021;16(10):e0257529. doi: 10.1371/journal.pone.0257529 34610022 PMC8491915

[31] González N, Marquès M, Nadal M, Domingo JL. Meat consumption: which are the current global risks? A review of recent (2010–2020) evidences. Food Research International. 2020;137:109341. doi: 10.1016/j.foodres.2020.109341 33233049 PMC7256495

[32] Taillie LS, Bercholz M, Prestemon CE, Higgins ICA, Grummon AH, Hall MG, et al. Impact of taxes and warning labels on red meat purchases among US consumers: A randomized controlled trial. PLoS Med. 2023;20(9):e1004284. doi: 10.1371/journal.pmed.1004284 37721952 PMC10545115

[33] National Cancer Institute. Cervical cancer screening. https://www.cancer.gov/types/cervical/screening. 2022. Accessed 2024 August 5

[34] Hornik RC, Yanovitzky I. Using Theory to Design Evaluations of Communication Campaigns: The Case of the National Youth Anti-Drug Media Campaign. Commun Theory. 2003;13(2):204–24. doi: 10.1111/j.1468-2885.2003.tb00289.x 25525317 PMC4267481

[35] Brewer NT, Parada HJ r, Hall MG, Boynton MH, Noar SM, Ribisl KM. Understanding why pictorial cigarette pack warnings increase quit attempts. Ann Behav Med. 2019;53(3):232–43. 29850764 10.1093/abm/kay032 PMC6265120

[36] Schiavo R. Health communication: from theory to practice. John Wiley & Sons. 2013.

[37] Pepper JK, Nguyen Zarndt A, Eggers ME, Nonnemaker JM, Portnoy DB. Impact of pictorial cigarette warnings compared with surgeon general’s warnings on understanding of the negative health consequences of smoking. Nicotine & Tobacco Research. 2020;22(10). doi: 10.1093/ntr/ntaa063 PMC10557086 32202624

[38] Williams Institute. Survey measures [Internet]. Available from: https://williamsinstitute.law.ucla.edu/quick-facts/survey-measures/

[39] United States Census Bureau. 2020 informational questionnaire. https://www2.census.gov/programs-surveys/decennial/2020/technical-documentation/questionnaires-and-instructions/questionnaires/2020-informational-questionnaire.pdf. 2020. Accessed 2025 September 22

[40] Centers for Disease Control and Prevention. Computer-assisted personal interview (CAPI) questionnaire: sexual behavior. National Health and Nutrition Examination Survey. https://wwwn.cdc.gov/nchs/data/nhanes/2019-2020/questionnaires/SXQ-CAPI-K-508.pdf. 2019. Accessed 2024 August 6

[41] Urban Institute. Health Reform Monitoring Survey. https://www.urban.org/policy-centers/health-policy-center/projects/health-reform-monitoring-survey/survey-resources. 2020. Accessed 2024 August 6

[42] Metro S. Metro SHAPE Adult Health Survey 2014: Ramsey County Data Book. https://www.ramseycounty.us/sites/default/files/Open%20Government/Public%20Health%20Data/ramsey_county_metro_SHAPE_2014_survey.pdf

[43] Kobayashi LC, Smith SG. Cancer Fatalism, Literacy, and Cancer Information Seeking in the American Public. Health Educ Behav. 2016;43(4):461–70. doi: 10.1177/1090198115604616 26377524 PMC5123630

[44] StataCorp. Stata Statistical Software: Release 18. College Station, TX: StataCorp. 2023.

[45] Musa J, Achenbach CJ, O’Dwyer LC, Evans CT, McHugh M, Hou L, et al. Effect of cervical cancer education and provider recommendation for screening on screening rates: A systematic review and meta-analysis. PLoS One. 2017;12(9):e0183924. doi: 10.1371/journal.pone.0183924 28873092 PMC5584806

[46] Uy C, Lopez J, Trinh-Shevrin C, Kwon SC, Sherman SE, Liang PS. Text Messaging Interventions on Cancer Screening Rates: A Systematic Review. J Med Internet Res. 2017;19(8):e296. doi: 10.2196/jmir.7893 28838885 PMC5590008

[47] Lorenc T, Petticrew M, Welch V, Tugwell P. What types of interventions generate inequalities? Evidence from systematic reviews. J Epidemiol Community Health. 2013;67(2):190–3. 22875078 10.1136/jech-2012-201257

[48] Malo TL, Gilkey MB, Hall ME, Shah PD, Brewer NT. Messages to Motivate Human Papillomavirus Vaccination: National Studies of Parents and Physicians. Cancer Epidemiol Biomarkers Prev. 2016;25(10):1383–91. doi: 10.1158/1055-9965.EPI-16-0224 27694109 PMC5108584

[49] Jeong M, Zhang D, Morgan JC, Ross JC, Osman A, Boynton MH. Similarities and differences in tobacco control research findings from convenience and probability samples. Ann Behav Med. 2019;53(5):476–85. 30052702 10.1093/abm/kay059 PMC6339836

[50] Peitzmeier SM, Khullar K, Reisner SL, Potter J. Pap test use is lower among female-to-male patients than non-transgender women. Am J Prev Med. 2014;47(6):808–12. doi: 10.1016/j.amepre.2014.07.031 25455121

[51] Kiran T, Davie S, Singh D, Hranilovic S, Pinto AD, Abramovich A, et al. Cancer screening rates among transgender adults: cross-sectional analysis of primary care data. Can Fam Physician. 2019;65(1):e30-7. PMC6347308 30674526

