# Supplementary material for: Perceived effectiveness of messages to address cervical cancer screening barriers: An online experiment
Source: PLoS One. 2025 Nov 14;20(11):e0336693. doi: 10.1371/journal.pone.0336693 (PMC12617949; doi:10.1371/journal.pone.0336693)
Supplement: S1 Table — Participants could select more than one response, so percentages do not total 100%. (DOCX) [file pone.0336693.s001.docx]

**Table S1.** Reasons for not being up to date with screening (n = 487)

|  | Never screened (n=318) | | Last screening more than 5 years ago (n=169) | |
| --- | --- | --- | --- | --- |
|  | n | % | n | % |
| I didn’t know that I am supposed to have routine CC screening | 108 | 34 | 35 | 21 |
| I am not sexually active | 77 | 24 | 40 | 24 |
| I have only 1 sexual partner | 55 | 17 | 29 | 17 |
| I am too busy | 51 | 16 | 19 | 11 |
| I have never heard of CC screening | 46 | 14 | N/A | N/A |
| I don’t want to know if something is wrong with my health | 38 | 12 | 22 | 13 |
| I have HPV vaccine | 23 | 7 | 8 | 5 |
| CC screening can’t prevent cervical cancer | 12 | 4 | 8 | 5 |
| Uninsured or too expensive | 6 | 2 | 24 | 14 |
| Other | 18 | 6 | 33 | 20 |

Participants could select more than one response, so percentages do not total 100%.
