# Supplementary material for: Perceived effectiveness of messages to address cervical cancer screening barriers: An online experiment
Source: PLoS One. 2025 Nov 14;20(11):e0336693. doi: 10.1371/journal.pone.0336693 (PMC12617949; doi:10.1371/journal.pone.0336693)
Supplement: S2 Table — Participants could select more than one response, so percentages do not total 100%. (DOCX) [file pone.0336693.s002.docx]

**Table S2.** Preferred channels for receiving messages about cervical cancer screening (n = 1,535)

|  | n | % |
| --- | --- | --- |
| Doctor | 1,100 | 72 |
| Social media | 749 | 49 |
| TV ads | 679 | 44 |
| Magazines, newspapers, billboards, or posters | 354 | 23 |
| Family | 328 | 21 |
| Other places | 39 | 3 |

Participants could select more than one response, so percentages do not total 100%.
