# Supplementary material for: Perceived effectiveness of messages to address cervical cancer screening barriers: An online experiment
Source: PLoS One. 2025 Nov 14;20(11):e0336693. doi: 10.1371/journal.pone.0336693 (PMC12617949; doi:10.1371/journal.pone.0336693)
Supplement: S3 Table — SD = standard deviation. (DOCX) [file pone.0336693.s003.docx]

**S3 Table.** Unadjusted primary and secondary outcomes for control message and by barrier-focused theme (n = 1,536)

|  | Perceived message effectiveness | Anticipated social interactions | Self-reported learning |
| --- | --- | --- | --- |
| Control  Mean (SD) | 2.45 (1.35) | 2.06 (1.32) | 1.96 (1.30) |
| Cancer Fatalism  Mean (SD)  Difference from control | 3.44 (1.21)  0.99 | 2.62 (1.37)  0.56 | 2.57 (1.34)  0.61 |
| Screening Guidelines  Mean (SD)  Difference from control | 3.44 (1.19)  0.99 | 2.63 (1.37)  0.57 | 2.92 (1.31)  0.96 |
| Risk Factors  Mean (SD)  Difference from control | 3.25 (1.23)  0.80 | 2.52 (1.37)  0.46 | 2.69 (1.33)  0.73 |
| Convenience  Mean (SD)  Difference from control | 3.43 (1.23)  0.98 | 2.60 (1.36)  0.54 | 2.53 (1.36)  0.57 |

SD = standard deviation.
