# Supplementary material for: Perceived effectiveness of messages to address cervical cancer screening barriers: An online experiment
Source: PLoS One. 2025 Nov 14;20(11):e0336693. doi: 10.1371/journal.pone.0336693 (PMC12617949; doi:10.1371/journal.pone.0336693)
Supplement: S4 Table — Associations are adjusted for arm in parent study (p = 0.168–0.372). Boldface indicates statistical significance (p < .05). (DOCX) [file pone.0336693.s004.docx]

**Table S4.** Adjusted impact of barrier-focused message theme on perceived message effectiveness, controlling for person-level characteristics (n = 1,483)

|  | *β* | SE | *p* |
| --- | --- | --- | --- |
| Message theme |  |  |  |
| Control (ref) | -- | -- | -- |
| Cancer fatalism | 1.01 | 0.03 | **.000** |
| Convenience | 0.99 | 0.03 | **.000** |
| Risk factors | 0.81 | 0.03 | **.000** |
| Screening guidelines | 1.01 | 0.03 | **.000** |
| Age (10 year increments) | -0.04 | 0.02 | **.042** |
| Race |  |  |  |
| White, non-Latine (ref) | -- | -- | -- |
| Black, non-Latine | 0.57 | 0.08 | **.000** |
| Latine | 0.24 | 0.08 | **.004** |
| Asian | 0.68 | 0.13 | **.000** |
| Another race or multiracial | -0.09 | 0.12 | .447 |
| Insured |  |  |  |
| No (ref) | -- | -- | -- |
| Yes | 0.17 | 0.08 | **.042** |
| Income |  |  |  |
| Less than $25,000 (ref) | -- | -- | -- |
| $25,000-49,999 | -0.09 | 0.07 | .239 |
| $50,000-99,999 | -0.08 | 0.07 | .259 |
| $100,000 or more | -0.11 | 0.09 | .205 |
| Sexual Orientation |  |  |  |
| Straight (ref) | -- | -- | -- |
| Lesbian, gay, bisexual, or another orientation | -0.07 | 0.08 | .387 |
| Cancer fatalism | -0.01 | 0.02 | .670 |
| Has a usual medical provider |  |  |  |
| No (ref) | -- | -- | -- |
| Yes | 0.29 | 0.07 | **.000** |
| Education |  |  |  |
| Less than or completed high school/GED (ref) | -- | -- | -- |
| Some College | -0.05 | 0.06 | .419 |
| Bachelors/Grad Degree | -0.18 | 0.06 | **.003** |
| Cervical cancer screening (past 5 years) |  |  |  |
| No (ref) | -- | -- | -- |
| Yes | -0.14 | 0.06 | **.020** |
| Self-reported health |  |  |  |
| Poor or fair (ref) | -- | -- | -- |
| Good | -0.04 | 0.06 | .493 |
| Very good/excellent | 0.10 | 0.07 | .132 |
| Baseline intentions to get screened |  |  |  |
| Definitely won’t (ref) | -- | -- | -- |
| Probably won’t | 0.45 | 0.13 | **.000** |
| Probably will | 0.88 | 0.11 | **.000** |
| Definitely will | 1.42 | 0.11 | **.000** |

Associations are adjusted for arm in parent study (*p* = 0.168-0.372). Boldface indicates statistical significance (*p*<.05).
